# A Case of Hypermotor Seizures in Posterior Insular Cortex Epilepsy

**DOI:** 10.7759/cureus.36280

**Published:** 2023-03-17

**Authors:** Dinesh M Chaudhari, Priyal ., Pushpendra Nath Renjen, Kamal Ahmad

**Affiliations:** 1 Neurosciences, Indraprastha Apollo Hospitals, New Delhi, IND; 2 Neurology, Indraprastha Apollo Hospitals, New Delhi, IND; 3 Neurology, Lady Hardinge Medical College, New Delhi, IND; 4 Internal Medicine, Indraprastha Apollo Hospitals, New Delhi, IND

**Keywords:** video eeg, focal seizure without impairment of awareness, medication-refractory epilepsy, insular epilepsy, insular cortex

## Abstract

Insular seizure is a rare entity. Insular spikes spread to the temporal, parietal, and frontal lobes and clinically manifest with seizure semiology specific to these areas. We report the case of a 19-year-old male patient who presented with complaints of left-sided hemimotor tonic-clonic focal seizures of the limbs occurring three times per day. Neuroimaging showed cortical-subcortical right posterior insular cortex hyperintensities on fluid-attenuated inversion recovery (FLAIR) sequence and T2-weighted MRI with no significant diffusion restriction on apparent diffusion coefficient (ADC) and no post-contrast enhancement, suggesting focal cortical dysplasia of right posterior insular cortex. Electroencephalogram (EEG) showed right frontal epileptiform activity with secondary bilateral synchrony. The patient's atypical hemimotor tonic-clonic focal seizure, the conventional video EEG showing right frontal spikes synchronizing with bilateral temporal ictal spikes, and insular cortical dysplasia on MRI led us to a diagnosis of insular epilepsy.

## Introduction

Insular epilepsy has a unique semiology among epilepsy disorders. Focal seizures are the most common clinical feature, and patients can present with either one or several types of focal seizures (focal tonic, focal tonic-clonic, and mydriasis associated with partial tonic-clonic seizures) [1-3]. Neuroimaging is usually unremarkable, and an electroencephalogram (EEG) can mimic frontal, parietal, or temporal seizures. Invasive EEG is diagnostic but associated with complications. We report a case of a 19-year-old male with insular cortex epilepsy diagnosed based on clinical features, neuroimaging, and EEG.

## Case presentation

A 19-year-old male presented to us with complaints of left-sided abnormal movements of the limbs, which occurred three times per day, each episode lasting for 10 minutes, and had persisted for the past nine years. These abnormal movements were not associated with loss of consciousness, bowel or bladder incontinence, or post-hoc confusion. The seizure first appeared 10 years ago, for which he had undergone neuroimaging and received anti-seizure medications. There was no history of febrile seizures, CNS infection, brain injury, brain surgery, and no family history of seizures.

**Figure 1:**
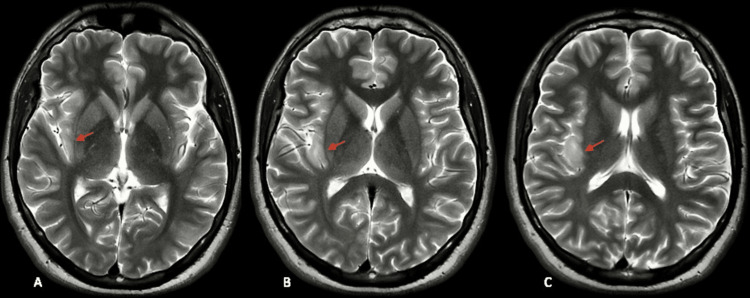
T2-weighted axial MRI images showing hyperintense right posterior insular focal cortical dysplasia MRI: magnetic resonance imaging

**Figure 2:**
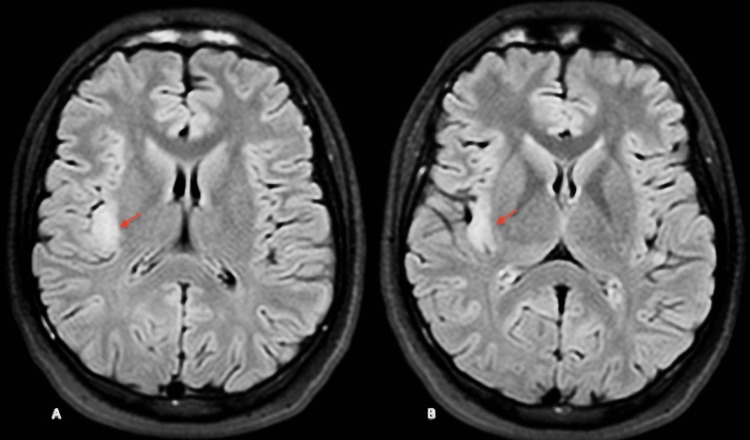
FLAIR sequence axial images showing right posterior insular focal cortical dysplasia FLAIR: fluid-attenuated inversion recovery

**Figure 3:**
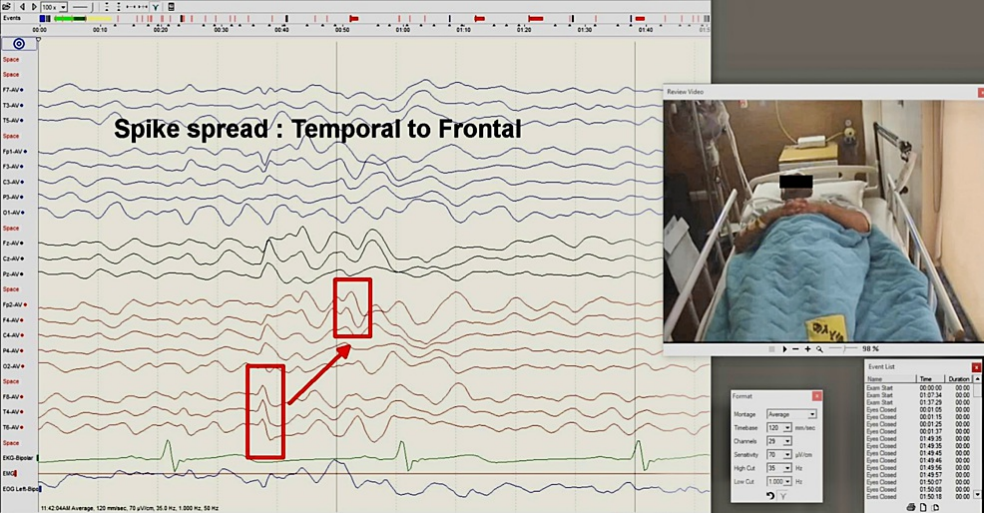
Right-sided temporal epileptiform discharges were seen on EEG, most active at F8-T4 The discharges were lateralized, with maximal amplitudes vacillating between the frontocentral, temporal, and centroparietal regions EEG: electroencephalogram

**Figure 4:**
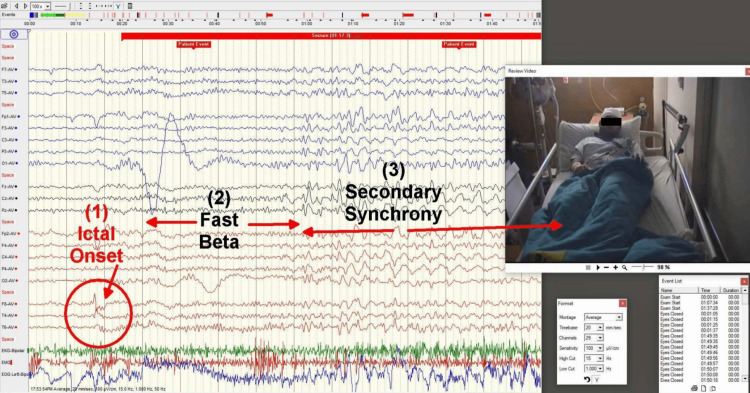
The surface EEG reveals an ictal-onset sharp wave from F8-T4, followed by diffuse 18 Hz beta activity for 5-8 seconds, then 3 Hz sharp-wave discharges during the TC phase, with decrescendo, abrupt offset, and postictal background suppression for 4-5 minutes The postictal phase is notable for more pronounced exhaustion in the right hemisphere compared to the left and the emergence of breakthrough spikes from F8-T4 EEG: electroencephalogram

On ictal examination, the patient was observed to have left-sided tonic-clonic movements. The cranial nerve examination, limb strength, deep tendon reflexes, and sensory system examination were all within normal limits. The planters were flexor, and pupils were equal and reactive to light. The Glasgow Coma Scale (GCS) score was 15. Cerebellar signs were negative. The patient was admitted for a video EEG. Routine labs were normal. Neuroimaging done two days prior to the admission showed a focal area of cortical and subcortical right posterior insular cortex hyperintensity on fluid-attenuated inversion recovery (FLAIR) sequence and T2-weighted MRI with no significant diffusion restriction on apparent diffusion coefficient (ADC) and no post-contrast enhancement, suggesting focal cortical dysplasia of right posterior insular cortex (Figures 1-2). EEG showed electroclinical seizures, stereotyped, each lasting about two minutes. The video revealed a brief awareness with a verbal indication of feeling unwell. In the next 20-30 seconds, this was followed by flapping or banging of the left arm, left limb extensor thrust dystonia, and rapid generalized tonic stiffness with a guttural cry. The head and torso rotated to the right side, with tonic-clonic movements for 90 seconds of the event. EEG showed right-sided frontal epileptiform activity with secondary bilateral synchrony (Figures 3-4). Routine MRIs done five and nine years back showed left temporoparietal encephalomalacia, perilesional gliosis, and hemosiderin.

The patient had been receiving levetiracetam 500 mg twice daily, which was stepped up to twice-daily dosing of 1 g of levetiracetam. He showed no improvement in seizures and 750 mg of valproic acid, 300 mg of oxcarbazepine, and 10 mg of clobazam were subsequently added to the treatment regime. The patient remained seizure-free on the follow-up EEG during the hospital stay and was discharged with follow-up advice.

## Discussion

The insula is buried inside the frontal, temporal, and parietal lobes, which prevents the EEG from reading epileptic insular nidus. As a result, on a conventional video EEG, distinct spike-wave synchrony on frontal and temporal electrodes is used to diagnose insular epilepsy aside from the patient's unique clinical features [4]. Insular epilepsy is hard to pick up on EEG and requires neuroimaging to support the diagnosis. Neuroimaging in insular epilepsy may show normal findings. But the characterization of the lesion of the insula (cavernous, cortical tubers, arteriovenous malformations, cystic encephalomalacia, Sylvian artery bifurcation aneurysm, glioma, focal cortical dysplasia such as polymicrogyria of insular gyri, and hypoplasia of insular lobules) before insular surgery determines the seizure-free outcome of insular resection since insular epilepsy is usually refractory to anti-epileptic drug therapy and may eventually require surgery [5-6].

The insular lobe is divided into an anterior and posterior lobule based on two factors: its functional connectivity with the rest of the brain and its unique seizure phenotype when either network is involved. The anterior lobule is divided from anterior to posterior into the anterior short gyrus, the middle short gyrus, and the posterior short gyrus. The posterior lobule is divided into a long anterior gyrus and a long posterior gyrus. A total of four to seven gyri are present, including normal population variants. Magnetic encephalogram (MEG) studies on insular functional connectivity during ictus have shown reproducible activation of the ipsilateral inferior frontal, orbitofrontal, mid-frontal, superior frontal, precentral gyrus, and mid-cingulate sulcus during anterior insular lobule seizures. The posterior lobule has neural interaction with the ipsilateral inferior angular, inferior supramarginal, caudal part of the superior temporal, and inferior postcentral gyri. A third inferior insular region was observed to be connected to the pregenual anterior cingulate cortex [7].

Correspondingly, anterior insular seizures cause hypermotor features (sudden, brief stereotypical movements such as kicking, thrashing, rocking, pelvic movements, bipedal and bimanual movements in sleep) and behavioral changes; and posterior insular seizures cause somatosensory features such as epigastric rising sensation, laryngeal constriction, hypersalivation, dysphonia, dysarthria, bradycardia, asystole, nausea, vomiting, piloerection, facial flushing, urge to urinate, the feeling of strangulation, and paresthesias (tactile, thermal, or painful) [8-9]. Seizures might not show up clinically until ictal activity leaves the insula, in which case the clinical phenotype matches the propagation pattern (especially spreading to the frontal lobe in hypermotor seizures). Perisylvian transmission of insular spikes, for example, presents with throat constriction, then progresses to perioral and hemi-somatosensory symptoms, and finally to unilateral ictal motor symptoms. Similarly, insular seizures may spread to the temporal, parietal, and frontal lobes and manifest in phenotypes specific to these areas [10]. However, insular epilepsy can coexist with primary frontal lobe epilepsy, temporal lobe epilepsy, or parietal lobe epilepsy, and it is hard to distinguish it from these disorders [9,11]. In the differential diagnosis, frontal, temporal, and parietal lobe epilepsy can be best ruled out based on clinical seizure phenotype; for example, ipsilateral manual automatism with déjà-vu is usually found in mesial temporal lobe epilepsy rather than insular epilepsy.

## Conclusions

We discussed a case of frontal propagation of posterior insular cortex epilepsy with hypermotor features (tonic-clonic seizures without impaired awareness). Insular seizures are uncommon entities. They are difficult to diagnose using standard video EEG and may require invasive EEG in life-threatening phenotypes (asystole and bradycardia). In all cases, radiography should be performed to screen for insular morphology. Early detection and treatment can help improve the quality of life of these patients.
